# Looking to the future through data: The rise and potential of gamification in medical education – A bibliometric analysis from 2000 to 2024

**DOI:** 10.1097/MD.0000000000046517

**Published:** 2025-12-12

**Authors:** Hua Xu, Yu-Nan Man, San-Mao Liu, Yu Sun, Lu-Yang Zhong, Mao-Lin He

**Affiliations:** a Education Evaluation and Faculty Development Center, Guangxi Medical University, Nanning, Guangxi Zhuang Autonomous Region, P.R. China; b Guangxi Collaborative Innovation Center for Biomedicine, Guangxi Medical University, Nanning, Guangxi Zhuang Autonomous Region, P.R. China (Guangxi-ASEAN Collaborative Innovation Center for Major Disease Prevention and Treatment, Guangxi Medical University, Nanning, Guangxi Zhuang Autonomous Region, P.R. China); c Division of Spinal Surgery, The First Affiliated Hospital of Guangxi Medical University, Nanning, Guangxi Zhuang Autonomous Region, P.R. China.

**Keywords:** bibliometrics, educational technology, gamification, medical education, visualization analysis

## Abstract

**Background:**

Advancements in educational technology have highlighted the significant potential of gamification in medical education. Herein we employed bibliometric methods to systematically analyze the development pathways and prospects of gamification in medical education from 2000 to 2024, offering data-driven insights for educators and researchers.

**Methods:**

Using the Web of Science Core Collection database, we identified 364 relevant articles on gamification in medical education published from 2000 to 2024 through a specific search strategy. Visualization analyses of publication trends, research hotspots, collaboration networks, and highly cited literature were performed using tools such as VOSviewer, CiteSpace, HisCite Pro, Bibliometrix, and ggplot2.

**Results:**

The analyzed literature spanned 796 institutions, with the United States and European countries leading in publication numbers and collaborative exchanges. The National University of Singapore made the most significant contributions, and Professor Tobias Raupach emerged as the most prolific and highly cited author. JMIR Serious Games was identified as the most influential journal in this field. Keyword clustering analysis indicated that teaching technologies in medical education are evolving from basic gamification towards more immersive, intelligent, and interactive learning experiences.

**Conclusion:**

Gamification enhances learner engagement and outcomes in medical education, fostering core medical competencies, and professional qualities. We report key development trends and research hotspots, emphasizing the importance of international collaboration. Future research should explore the long-term benefits of gamified learning, personalized learning paths, and the integration of gamification within ethical and standard frameworks to drive continuous innovation in medical education.

## 1. Introduction

Considering the rapid evolvements in the medical field, effective education and training are essential for improving healthcare quality. Traditional medical education models, while systematic and comprehensive, often fail to adequately stimulate learner motivation and engagement.^[1,2]^ In recent years, technological advancements have brought gamification and game-based learning to the forefront, demonstrating significant potential in medical education.^[3,4]^ Gamification involves integrating game design elements and mechanics into non-game contexts to enhance engagement, motivation, and learning outcomes. This approach not only makes learning more enjoyable but also simulates real-world challenges, thereby enriching the educational experience.^[5,6]^

Gamification holds immense potential in medical education due to its ability to create interactive and immersive learning environments that foster active participation and practical skill enhancement. For instance, gamified training simulations, such as patient management, virtual surgery, or emergency response scenarios, enable learners to practice and refine clinical skills in a risk-free environment. Moreover, gamified education provides personalized learning pathways through real-time feedback and assessment, helping learners identify their strengths and areas for improvement.^[7,8]^ Research demonstrates that gamification significantly enhances learner motivation and academic performance. Compared to traditional methods, gamified learning fosters competition and a sense of achievement through game mechanics, effectively increasing engagement.^[9]^

In medical education, gamification not only strengthens knowledge acquisition but also improves practical skills through real-world simulations and challenge-based learning. In addition, gamification collaborative game tasks promote teamwork and communication by allowing learners to practice these skills in virtual settings.^[10–13]^ Integrating gamified learning into medical education enables learners to achieve significant progress in knowledge acquisition, skill application, and practical performance, enhancing overall educational efficiency.^[14]^

Bibliometrics, extensively employed across various research disciplines, systematically uncovers academic development trends, evaluates scholarly impact, traces research evolution, and identifies key researchers and collaboration networks.^[15]^ Given the substantial potential of gamification in medical education and gaps in the current knowledge framework, bibliometric methods provide a systematic approach for identifying emerging trends and research priorities. Further, this approach establishes a strong foundation for academic discussions and practical applications, advancing interdisciplinary research and practice.

## 2. Methods

### 2.1. Search strategy, inclusion, and exclusion criteria

A comprehensive literature search was performed using the Web of Science Core Collection (WOSCC) database to identify relevant studies on gamification in medical education. The following Boolean query was employed: (((TS=(“medical education” OR “medical teaching” OR “medical training” OR “health education” OR “clinical education” OR “nursing education” OR “healthcare education”)) AND TS = (Gamification OR “Game-based learning” OR “serious game” OR “Gamified learning”)) AND DT = (Article OR Review)) AND LA = (English).

The search was confined to publications from January 1, 2000, to November 13, 2024. Articles and reviews were included, while commentaries, letters, conference abstracts, and retracted publications were excluded.^[16]^ Only English-language documents were considered. Two authors (Xu Hua and Man Yunan) independently performed data retrieval and screening using the WOSCC database, with all selected records exported in BibTeX. Discrepancies were resolved by a third unbiased author (Liu San-Mao) to ensure consistency and objectivity in the selection process. Ultimately, 364 articles meeting the inclusion criteria were incorporated into the bibliometric analysis.

### 2.2. Harmonization of keywords

The initial data generated by WOSCC was preprocessed to increase its robustness prior to bibliometric network analysis:

#### Merging keyword synonyms

Semantically related phrases (e.g., “game-based learning” → “gamified learning”; “virtual reality” → “VR”; “medical student” → “students”) were merged into standardized nodes during co-word analysis using VOSviewer.

#### Author name disambiguation

Disparities in author names (e.g., “Tobias Raupach” vs “T, Raupach”) were clarified by comparing publication histories, institutional affiliations, and ORCID profiles. The papers of Tobias Raupach from the University of Göttingen, for example, were all grouped together under 1 identification.

### 2.3. Research methods

**Figure 1.**
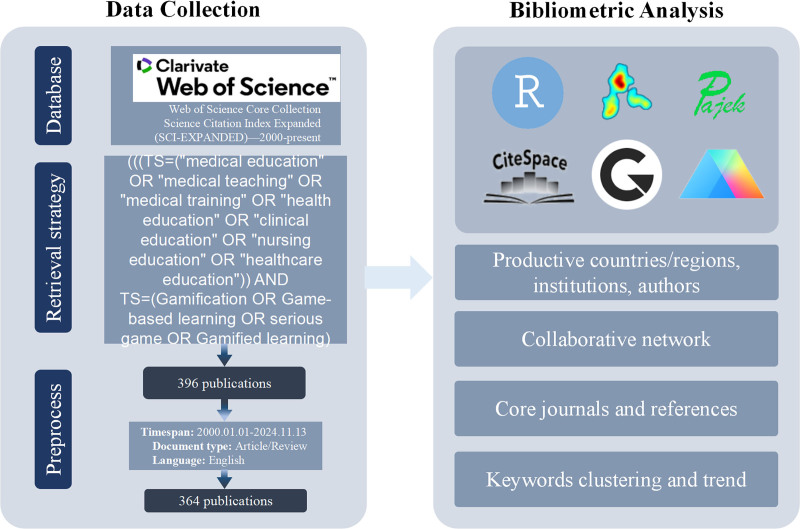
Retrieval and analysis flowchart.

Bibliometric analyses were performed using VOSviewer v1.6.18 (Centre for Science and Technology Studies [CWTS], Leiden University, Leiden, The Netherlands), CiteSpace v6.1.3 (College of Computing and Informatics, Drexel University, Philadelphia), HisCite Pro 2.1 (SciTech Strategies, Inc., Philadelphia), the Bibliometrix package in R v4.2.1 (R Foundation for Statistical Computing, Vienna, Austria), ggplot2 v3.5.1, and GraphPad Prism v9.5.1 (GraphPad Software, LLC [a Dotmatics company], Boston). VOSviewer was utilized to create literature-related network maps and heatmaps, as well as for co-word and coupling analyses to illustrate trends in gamified learning in medical education.^[17]^ CiteSpace and Bibliometrix were employed to visualize knowledge maps, analyze research trends and hotspots, cluster literature, and identify key publications and authors.^[18,19]^ HisCite Pro 2.1 was used to quantify and analyze total publication volume and citation counts, identify highly cited studies, and visualize annual publication trends.^[20]^ Lastly, ggplot2 and GraphPad Prism were applied to visually present the findings. Figure 1 shows our research workflow.

## 3. Results

### 3.1. Overall description

**Figure 2.**
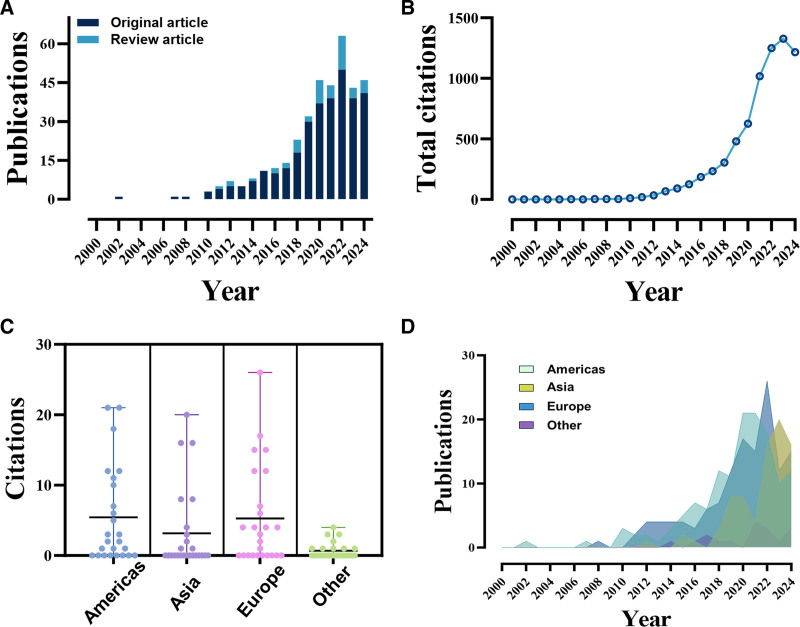
Comprehensive overview of bibliometric analysis. (A) Annual publication volume by article type. (B) Annual citation volume. (C) Citation volume across continents (Bee Swarm Plot): represents the distribution of citationvolumes across various continents using a bee swarm visualization. Others: Oceania and Africa. (D) Annual publication volume by continent (stacked plot). Others: Oceania and Africa.

After conducting a comprehensive search within the WOSCC database and excluding studies that did not meet our inclusion criteria, 364 articles were ultimately included in our analyses. These studies were published from 2000 to 2024 and involved contributions from 1782 authors affiliated with 796 distinct institutions. The articles were disseminated across 140 different academic journals. Annual citation counts, citation volumes of individual articles, and yearly number of publications across various continents showed a consistent upward trend, reflecting growing research interest and sustained attention to the study topic (Fig. 2 and Table S1, Supplemental Digital Content, https://links.lww.com/MD/Q887).

### 3.2. Global collaboration and development trends

Global collaboration has been instrumental in advancing medical education research. Countries are increasingly sharing teaching resources and best practices through international partnerships and exchanges, promoting a transition from regional or national standards to unified global benchmarks. This shift fosters the standardization and internationalization of medical education.^[21]^ Consequently, one of our primary objectives was to elucidate the development trends in medical education and gamification across various countries over the past 2 decades.

**Figure 3.**
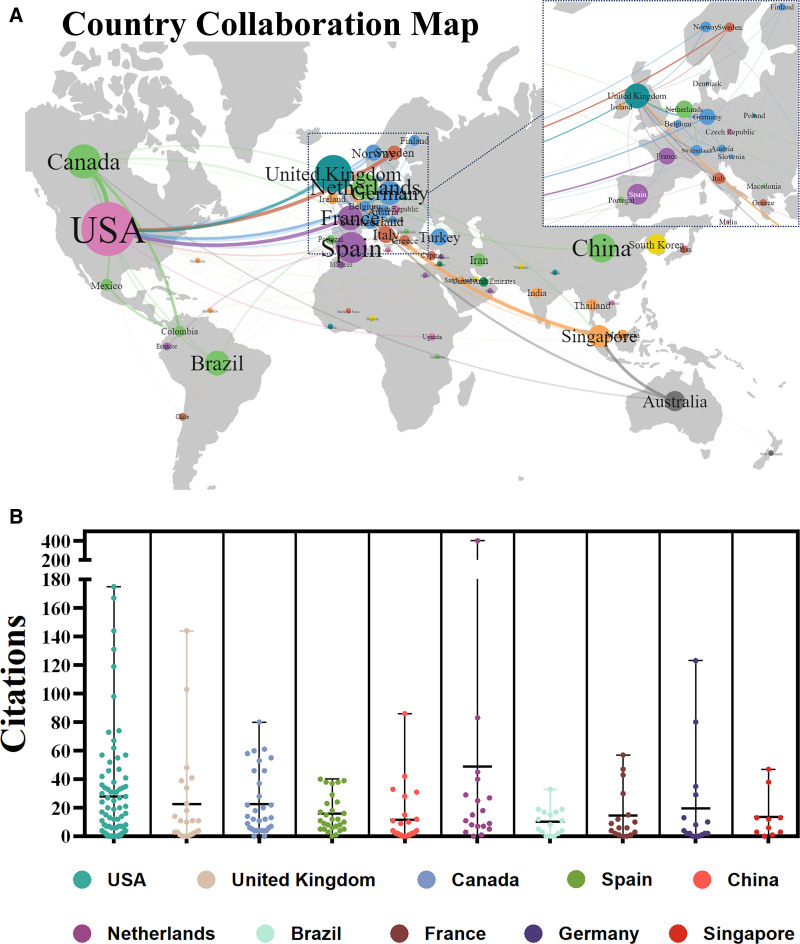
Global collaboration and citation impact. (A) National Collaboration Map. (B) Bee Swarm Plot of citation counts for the top 10 publishing countries.

The United States was found to lead the field, contributing 27.19% of total publications and amassing 2298 citations, thereby maintaining a dominant position. The United Kingdom (47 publications), Canada (40), Spain (33), and China (28) were next in terms of publication volume. Notably, despite having fewer publications, the Netherlands ranked second to the United States in total citation counts (Fig. 3 and Table 1). Furthermore, the country collaboration map illustrated extensive collaborative exchanges between European nations and the United States. While Singapore demonstrated strong collaborative exchanges with Italy, the extent of cooperation among other Asian countries significantly varied. Although several Asian nations have made noteworthy advancements in promoting transnational collaborations, considerable potential remains for further enhancing international cooperation.

**Table 1:** The top 10 countries/regions with the highest productivity.

Rank	Country	Publications n (%)	Total citations	Average citations	Collaborative centrality
1	USA	99 (27.19%)	2298	23.21	0.48
2	United Kingdom	47 (12.91%)	684	14.55	0.63
3	Canada	40 (10.98%)	800	20.00	0.22
4	Spain	33 (9.06%)	572	17.33	0.09
5	China	28 (7.69%)	365	13.04	0.08
6	Netherlands	23 (6.31%)	898	39.04	0.02
7	Brazil	21 (5.76%)	167	7.95	0.15
8	France	21 (5.76%)	230	10.95	0.03
9	Germany	20 (5.49%)	396	19.80	0.02
10	Singapore	17 (4.67%)	361	21.24	0.07

Limited international collaboration risks knowledge isolation, perpetuating repetitive methodologies and perspectives within confined environments, which can hinder innovation and the diversity of research approaches. Our findings visually highlight the imperative of strengthening international cooperation to foster innovation and advancement through comprehensive data analysis.

### 3.3. Collaboration between institutions (Universities) and authors

Among the 796 institutions included in this study, the National University of Singapore ranked 1st in both the number of publications and total citations, surpassing leading institutions from the United States and Europe. This highlights its exceptional performance and leadership in advancing gamification in medical education. Other top-ranking institutions included Harvard Medical School, the University of Alberta, the University of Toronto, and Imperial College London (Table 2).

**Table 2:** The top 10 productive institutions.

Rank	Institution	Country	Publicationsn (%)	Total citation	Average citation
1	National University of Singapore	Singapore	11 (3.02%)	220	20.00
2	Harvard Medical School	USA	7 (1.92%)	86	12.29
3	University of Alberta	Canada	7 (1.92%)	128	18.29
4	University of Toronto	Canada	7 (1.92%)	169	24.14
5	Imperial College London	United Kingdom	6 (1.64%)	139	23.17
6	Nanyang Technological University	Singapore	6 (1.64%)	140	23.33
7	The National University Hospital	Singapore	6 (1.64%)	145	24.17
8	Stanford University	USA	6 (1.64%)	122	20.33
9	Columbia University	USA	5 (1.37%)	184	36.80
10	Universidad de Almería	Spain	5 (1.37%)	72	14.40

**Figure 4.**
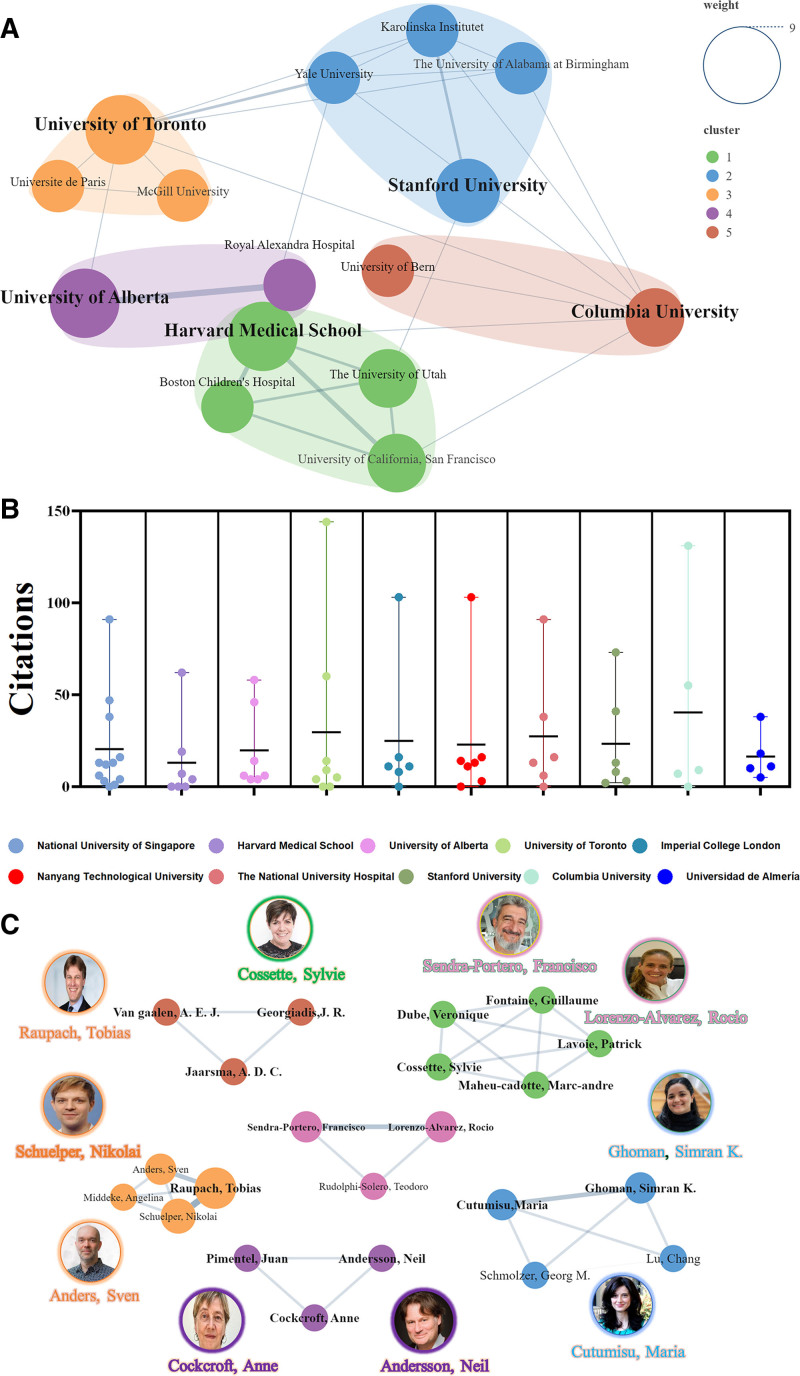
Collaboration analysis of institutions and authors. (A) Cluster analysis of collaborations among institutions. (B) Total citation counts for each institution. (C) Cluster analysis of collaborations among authors.

Utilizing VOSviewer, a cluster analysis of collaborations among 25 institutions with > 4 publications revealed the dominant roles of Harvard Medical School, Stanford University, the University of Toronto, and Columbia University (Fig. 4A). Citation counts were also visualized for the top 10 institutions (Fig. 4B).

Collaboration analyses of 22 authors with > 3 publications identified Professor Tobias Raupach (University of Göttingen) as the most prolific author with the highest output and citation counts. Among the top 10 authors, 5 were from Europe and the remainder from Canada, with no representation from Asia (Table 3). On visualizing collaborative relationships among these authors (Fig. 4C), we realized that coauthor clusters were predominantly concentrated within the same institution or country, reflecting a scarcity of cross-national or cross-institutional collaboration. This pattern may explain why, despite its publication dominance, the National University of Singapore has not assumed a leading role in international collaborations. The lack of extensive international cooperation may constrain its global influence and collaborative network, underscoring the need for cross-national and cross-institutional collaborations to enhance internationalization, and research impact.

**Table 3:** Top 10 productive authors.

	Author	Institution	Country	Publications n (%)	Total citation	Average citation	*H*-index
1	Raupach, Tobias	University of Gottingen	Germany	7 (1.92%)	99	14.14	28
2	Schuelper, Nikolai	Medius Klin Ostfildern Ruit	Germany	5 (1.37%)	97	19.40	9
3	Anders, Sven	University of Alberta	Canada	4 (1.09%)	62	15.50	18
4	Cutumisu, Maria	McGill University	Canada	4 (1.09%)	66	16.50	15
5	Ghoman, Simran K.	Royal Alexandra Hospital	Canada	4 (1.09%)	66	16.50	5
6	Lorenzo-Alvarez, Rocio	Hosp Serrania	Spain	4 (1.09%)	84	21.00	6
7	Sendra-Portero, Francisco	Universidad de Malaga	Spain	4 (1.09%)	84	21.00	11
8	Andersson, Neil	McGill University	Canada	3 (0.82%)	11	3.67	33
9	Cockcroft, Anne	McGill University	Canada	3 (0.82%)	11	3.67	21
10	Cossette, Sylvie	Institut de Cardiologie de Montreal	Canada	3 (0.82%)	95	31.67	22

### 3.4. Identification of core journals

**Figure 5.**
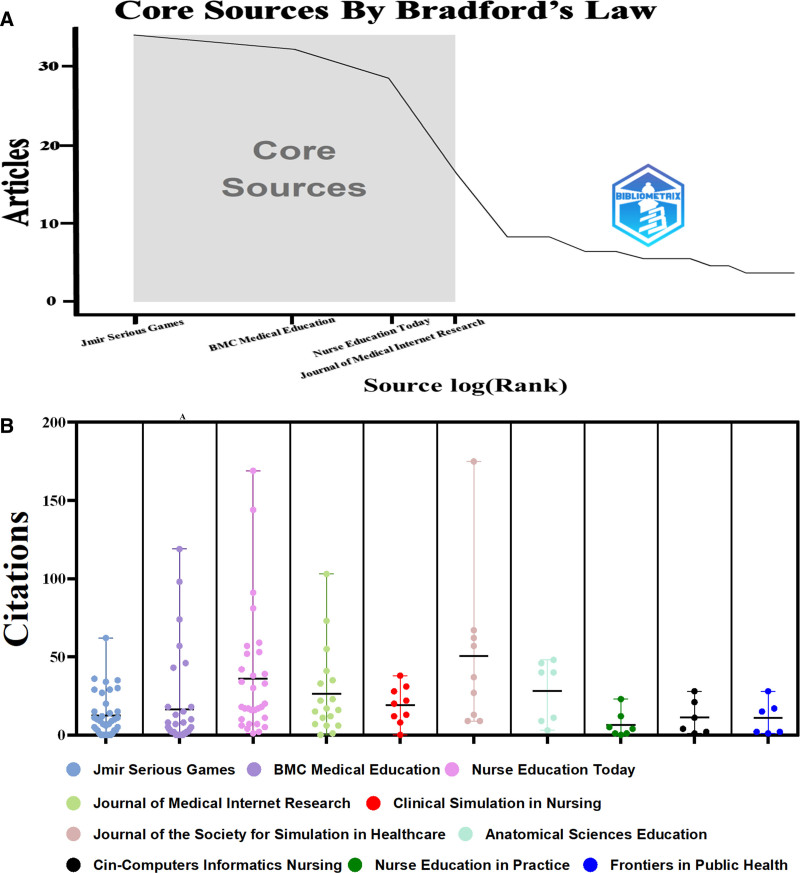
Analysis of journals. (A) Core journals identified by Bradford law using the bibliometrix package. (B) Bee Swarm Plot of article citations for the top 10 journals by publication volume.

Within the domain of medical education and gamification, we identified 140 journals. Utilizing Bradford law for source clustering analysis, core journals were determined (Fig. 5A).^[22]^ This analysis identified journal clusters with the highest number of relevant publications, pinpointing the most important and influential journals in the field.

Tables 4 and 5 present pertinent basic information, JCR category quartiles, impact factors, and the top 10 most cited articles of the 10 leading journals. Approximately 45.3% of articles on medical education and gamification were published in these journals, primarily within the fields of medical education and nursing. The majority were Q1 journals, with JMIR Serious Games leading in publication volume and the Journal of Medical Internet Research possessing the highest impact factor. Six journals had impact factors > 3, reflecting their substantial citation rates and recognition within the academic community. Citation profiles of these 10 journals were visualized for an intuitive representation of their influence (Fig. 5B).

**Table 4:** Top 10 core journals.

Rank	Journal	Publications n (%)	Total citations	Average citations	2022 JCR category quartile	2022 IF
1	Jmir Serious Games	37 (10.16%)	429	11.59	Q1	4.0
2	BMC Medical Education	35 (9.61%)	506	14.46	Q1	3.6
3	Nurse Education Today	31 (8.51%)	956	30.84	Q1	3.9
4	Journal of Medical Internet Research	18 (4.94%)	436	24.22	Q1	7.4
5	Clinical Simulation in Nursing	9 (2.47%)	152	16.89	Q1	2.6
6	Journal of the Society for Simulation in Healthcare	9 (2.47%)	406	45.11	Q3	2.4
7	Anatomical Sciences Education	7 (1.92%)	180	25.71	Q1	7.3
8	Nurse Education in Practice	7 (1.92%)	42	6.00	Q1	3.2
9	Cin-Computers Informatics Nursing	6 (1.64%)	63	10.50	Q3	1.3
10	Frontiers in Public Health	6 (1.64%)	58	9.67	Q2	5.2

IF = Impact Factor, JCR = Journal Citation Reports.

**Table 5:** Ten core literatures with the highest citations in the field of gamification in medical education.

Rank	First author	Title	Journal	Type	Year of publication	Total citations
1	Graafland, Maurits	Systematic review of serious games for medical education and surgical skills training	British Journal of Surgery	Review	2012	401
2	Van Gaalen, A. E. J.	Gamification of health professions education: a systematic review	Advances in Health Sciences Education	Review	2021	194
3	Gorbanev, Iouri	A systematic review of serious games in medical education: quality of evidence and pedagogical strategy	Medical Education Online	Review	2018	180
4	Wang, Ryan	A Systematic Review of Serious Games in Training Health Care Professionals	Simulation in Healthcare	Review	2016	175
5	Nevin, Christa R.	Gamification as a tool for enhancing graduate medical education	Postgraduate Medical Journal	Article	2014	167
6	Gomez-Urquiza, Jose L.	The impact on nursing students’ opinions and motivation of using a Nursing Escape Room as a teaching game: A descriptive study	Nurse Education Today	Article	2019	169
7	Rutledge, Chrystal	Gamification in Action: Theoretical and Practical Considerations for Medical Educators	Academic Medicine	Article	2018	144
8	Wiemeyer, J	Serious games in prevention and rehabilitation-a new panacea for elderly people?	Computers & Education	Review	2012	123
9	Cant, Robyn P.	Simulation in the Internet age: The place of Web-based simulation in nursing education. An integrative review	Nurse Education Today	Review	2014	144
10	Kron, Frederick W.	Medical student attitudes toward video games and related new media technologies in medical education	Computers & Education	Article	2010	119

### 3.5. Evolution of research hotspots and future directions

**Figure 6.**
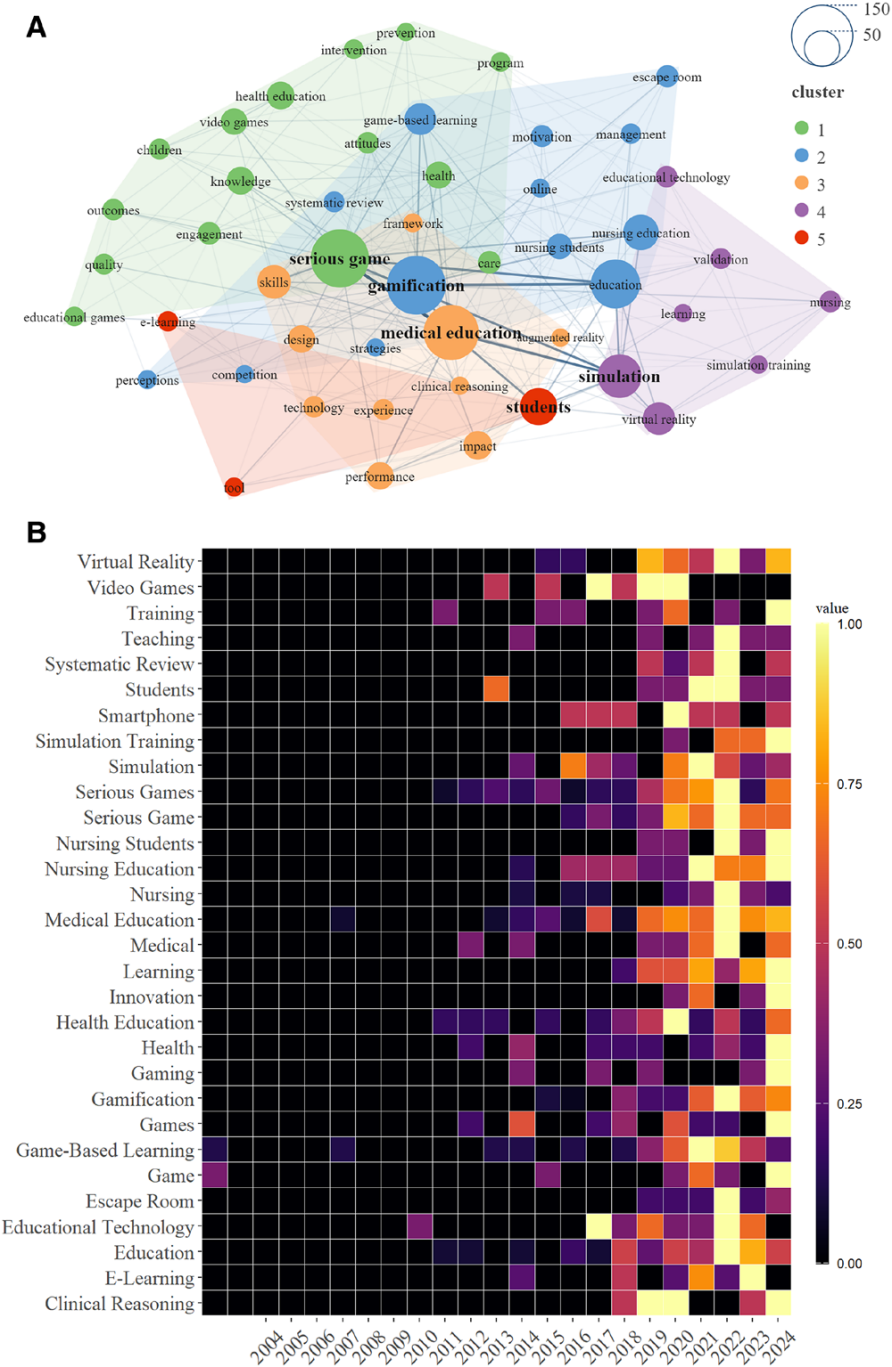
Analysis of keywords. (A) 48 keywords with a frequency exceeding 9 were categorized into 5 distinct clusters. (B) Heat map of keywords.

Keyword analysis highlighted research hotspots and trends, evaluated research impact, identified gaps, and optimized resource allocation, facilitating comprehensive field advancement. A clustering analysis of 48 keywords with a frequency of > 9 revealed a strategic map of research hotspots. Keywords with similar meanings were grouped into the same cluster. Clusters 1 (green) and 2 (blue) predominantly focused on diverse strategies such as health interventions and outcome evaluations for enhancing learning effectiveness. Cluster 3 (yellow) accentuated skill training and innovative teaching methodologies. Cluster 4 (purple) focused on the application and validation of simulation training and virtual reality (VR) applications in medical and nursing education. Lastly, Cluster 5 (red) focused on the utilization and development of e-learning tools in student education (Fig. 6A).

A time-evolving keyword heatmap revealed a minimal emphasis on gamified education during the early 2000s, followed by a marked surge in interest in recent years. Keywords evolved from basic terms such as “game” and “game-based learning” to more sophisticated concepts such as “smartphone,” “video games,” and “virtual reality,” reflecting the progression of teaching technologies in medical education. This transition underscores the ongoing evolution and diversification of educational technology applications (Fig. 6B).

**Figure 7.**
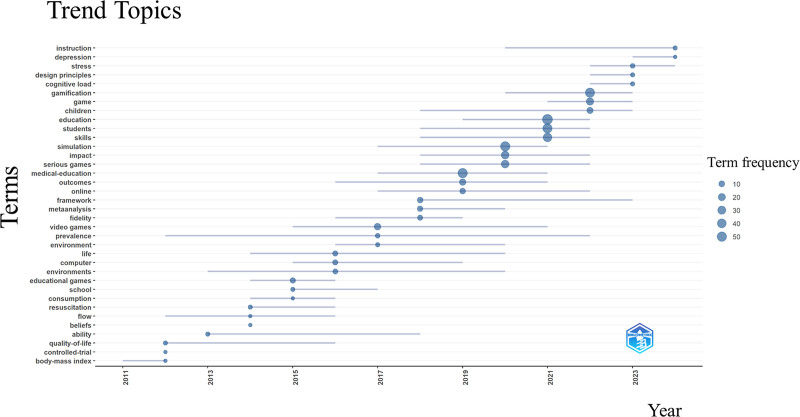
Trend topics from 2000 to 2024.

Leveraging Bibliometrix, we analyzed the evolution of research topics in medical education and gamification through annual variations, which largely mirrored the observed keyword trends (Fig. 7). In recent years, the emergence of keywords such as “depression,” “stress,” and “cognitive load” highlights a growing concern among researchers regarding the impact of medical education and learning environments on the psychological well-being and cognitive states of students and educators. It is widely recognized that stress, depressive emotions, and cognitive load during the learning process significantly influence student performance and overall educational outcomes. Consequently, alongside the advancement of educational technologies and teaching methodologies, ensuring that students maintain psychological health and cognitive balance while achieving effective learning outcomes has become pivotal.

In this context, gamified learning has emerged as an innovative educational paradigm, garnering extensive attention and application. By integrating gamification elements (e.g., challenges, rewards, interactive feedback, and role-playing) into the learning process, gamified learning can not only enhance student motivation and engagement but also effectively mitigate learning-related stress and anxiety through its immersive and interactive nature.

## 4. Discussion

### 4.1. General information

The field of gamified learning in medical education began to take shape around 2002. However, its initial development progressed at a measured pace, likely due to researchers adapting to this novel domain and the requisite time needed to collect and analyze extensive educational data. Beginning in 2018, with the formalization of gamification principles, research in this field entered a period of rapid expansion, marked by a significant surge in study volume.^[23,24]^ Dr Gorbanev et al identified key challenges in applying gamification in medical education, including the absence of rigorous design and evaluation methodologies, as well as limited dissemination. To address these gaps, their team focused on developing specialized MeSH terms to refine and enhance teaching strategies.^[25]^ By 2022, the field reached a new pinnacle, closely linked to technological advancements, notably the widespread adoption of VR. These advancements propelled medical education toward more dynamic and interactive modalities, providing realistic challenges and feedback to enhance learner engagement and educational outcomes.

In summary, gamified learning demonstrates immense potential and offers substantial development prospects within medical education. Based on the findings of this study, attention should focus on effectively integrating gamification principles into various aspects of medical education. In particular, attention should be directed toward the following areas.

### 4.2. Multidimensional impact of gamified learning on medical education

Gamified learning in medical education impacts not only student engagement and learning outcomes but also cognitive development and emotional well-being. By incorporating interactive elements and game mechanics, this approach stimulates cognitive abilities, promoting critical thinking, and problem-solving skills. While this approach enhances motivation and engagement, it also presents challenges.

From a cognitive perspective, gamified learning leverages interaction and feedback mechanisms to foster problem-solving abilities.^[26]^ For instance, tasks and challenges within games often require students to engage in complex thinking and decision-making processes, deepening their learning and cultivating clinical judgment and decision-making capabilities.^[27]^ However, it may also increase cognitive load, particularly if tasks are overly complex. Emotional benefits, such as a sense of achievement and social interaction, can enhance persistence and memory retention, yet high-intensity tasks may elevate stress levels, potentially hindering learning outcomes.

Emotionally, gamified learning fosters positive experiences, such as a sense of achievement and social interaction. These can enhance learning persistence and memory retention. For example, completing tasks or earning rewards in games increases intrinsic motivation and engagement.^[28]^ Moreover, the social elements within games encourage peer interaction and collaborative problem-solving, fostering teamwork, and communication skills.^[29]^ However, these benefits depend on the careful design of game mechanics to ensure inclusive and equitable participation.

### 4.3. Integration of personalized learning paths with gamified education

Personalized learning tailors educational content and pacing to the needs, interests, and learning styles of individual students. Gamified education, with its inherent interactivity, enriches this experience. Combining these approaches offers a more flexible and impactful learning experience.

First, personalized learning paths allow students to progress at their own pace within a gamified environment. By assessing baseline knowledge and learning preferences, educators can design customized tasks and challenges that boost confidence and engagement, thereby motivating students. Second, the feedback mechanisms in gamified education enable dynamic adjustments to learning strategies. Immediate feedback helps students refine their approach,^[30]^ while educators can use performance data to adapt learning content and teaching methods. Finally, integrating student interests and career aspirations into gamified education makes learning more relevant and engaging. However, this raises concerns about fairness in resource allocation and assessment standards. For instance, students with different baseline skills or technological access may experience unequal learning opportunities. Future designs should aim to balance personalized flexibility with standardized evaluation frameworks to ensure equity.

### 4.4. Long-term benefits and sustainability of educational outcomes

While gamified learning significantly enhances learning motivation and short-term academic performance, its impact on long-term educational outcomes and professional development remains unclear.^[31]^ Future research should thus focus on evaluating its role in cultivating core medical competencies and professional qualities.

Core competencies, such as clinical skills, communication, and teamwork, are indispensable for healthcare professionals. Gamified learning may refine these skills through real-world simulations. Besides, research should explore how this approach cultivates professional qualities, including ethics and stress management, through longitudinal studies. Finally, whether gamified learning fosters lifelong learning habits is a key consideration. Encouraging students to reflect on their learning processes within games may promote adaptability and continuous education throughout their careers.

### 4.5. Ethical and standard challenges of gamified learning in medical education

Despite its advantages, gamification poses challenges to educational ethics and standards, particularly when virtual decision-making diverges from real-world ethical standards.^[32]^ For example, simulated medical procedures may raise concerns about alignment with real-world ethical standards. Addressing these issues requires integrating ethical norms into game design to prevent misconceptions or inappropriate decision-making.

### 4.6. Interpretation of emerging trends and practical translation

The bibliometric trends toward immersive technology (like VR/augmented reality) and psychological aspects (like stress, cognitive load) are critical responses to the challenges in medical education. Accreditation Council for Graduate Medical Education standards that use VR’s objective metrics, regulatory shifts toward competency-based evaluations, pedagogical demands for safe high-risk skill training (like surgery), and declining technology costs that allow for scalable high-fidelity simulations are some of the reasons behind the growth of VR.^[1]^ However, there is now more focus on psychological well-being due to evidence of learner burnout and the realization that gamification must balance engagement and ethical design to lessen cognitive overload^[2]^.

Therefore, policymakers need to fund adaptive systems that customize cognitive load and mandate psychological impact assessments for gamified tools. Teachers should give priority to co-design mechanisms that reduce competition-induced stress and accessible implementations. To standardize learner-centric innovation, stakeholders should collaborate to develop global guidelines. This will guarantee that innovations in technology, such as VR, bridge skill-transfer gaps while preserving mental health via moral protections.

### 4.7. Future research directions

To address the limitations identified, future research should:

- Explore innovative game designs that integrate educational goals with engaging and intuitive mechanics.
- Investigate the psychological impact of gamified learning, with a focus on reducing cognitive load and stress.
- Conduct longitudinal studies to evaluate the sustainability of learning outcomes and their impact on professional development.
- Strengthen interdisciplinary and international collaborations to promote diverse perspectives and best practices in gamified learning.

In conclusion, gamified learning represents an innovative effort to enhance learning outcomes while prioritizing the psychological well-being of students. This suggests that future educational methodologies will prioritize flexibility and personalization to provide students with a highly supportive, interactive, and positive learning environment.

## 5. Limitations

Although this study provides significant insights into research hotspots and development trends in gamified learning in medical education, several limitations warrant further consideration.

### Database limitation

The fact that this study solely relied on the WOSCC database should be recognized as a flaw. WOSCC is a premier source for high-impact scientific literature and provides trustworthy citation information required for bibliometric mapping and network analysis, but its coverage is not comprehensive. This dependence may introduce biases related to the comprehensiveness of the data. For instance, relevant research may have been missed in journals not in WOSCC’s index, particularly regional journals, some specialized journals devoted to educational technology or game design, or more recent open-access publications. Furthermore, because WOSCC places a strong emphasis on English-language publications, it might underrepresent significant research conducted and published in other languages. As a result, while the WOSCC-indexed literature captures the essence of the field’s development in mainstream academic publishing, our findings largely reflect the patterns and collaborations found there, which may not accurately reflect the full scope of global research on gamification in medical education, particularly in non-Anglophone contexts or emerging platforms.

### Impact of timespan

Although we included literature from 2000 to 2024, the focus and methodologies employed in research may have varied across different periods. Early studies may not have fully embraced gamified learning principles, while more recent research may be influenced by emerging technologies, such as VR. These temporal variations complicate direct comparisons of research outcomes across different timeframes.

### Methodological diversity

Research on gamified learning employs various methodologies, including quantitative, qualitative, and mixed-method approaches. This diversity can hinder the comparability of results, affecting a comprehensive understanding of overall research trends. In addition, variations in study design and implementation quality may impact data validity and reliability.

### Cultural and regional differences

International collaboration and exchange, as highlighted in the study, may be influenced by cultural and regional factors. Different countries and regions exhibit varying practices and levels of acceptance regarding gamified learning. Such disparities can limit the generalizability and global applicability of research findings.

## 6. Conclusions

Herein we performed a systematic bibliometric analysis of the literature on gamified learning in medical education, thoroughly elucidating key research hotspots and development trends. Gamified learning exhibits significant potential in enhancing student engagement and short-term academic performance while playing a pivotal role in cultivating core medical competencies and professional qualities. However, the long-term benefits and sustainability of gamified learning remain underexplored, presenting extensive opportunities for future research.

Keyword analysis revealed a shift in recent research focus from foundational concepts of gamified learning to more complex themes, such as psychological health, cognitive load, and immersive learning experiences. This transition underscores the growing emphasis of the academic community on the interconnection between the psychological well-being of students and their learning outcomes in medical education. Moreover, promoting cross-national and cross-institutional collaborations is particularly essential. Enhanced international knowledge-sharing and resource integration could enable the adoption of best practices from diverse regions, driving innovation and advancement in gamified learning.

To conclude, gamified learning holds immense potential to enrich and diversify teaching methodologies in medical education. By fostering adaptable and professionally competent healthcare professionals, this approach equips them to address future challenges and seize opportunities within the dynamic medical field.

## Acknowledgments

The authors thank all the public data sources involved in the present study.

## Author contributions

**Conceptualization:** Yu Sun, Mao-Lin He.

**Data curation:** Hua Xu, Yu-Nan Man, San-Mao Liu.

**Investigation:** Hua Xu.

**Project administration:** Hua Xu, Mao-Lin He.

**Resources:** Hua Xu, San-Mao Liu.

**Software:** Yu-Nan Man, Lu-Yang Zhong.

**Supervision:** Mao-Lin He.

**Validation:** Hua Xu, Yu Sun.

**Visualization:** Hua Xu, Yu-Nan Man.

**Writing – original draft:** Hua Xu, Yu-Nan Man, San-Mao Liu, Yu Sun, Lu-Yang Zhong.

**Writing – review & editing:** San-Mao Liu, Mao-Lin He.

## Supplementary Material
