# Supplementary material for: Looking to the future through data: The rise and potential of gamification in medical education – A bibliometric analysis from 2000 to 2024
Source: Medicine (Baltimore). 2025 Dec 12;104(50):e46517. doi: 10.1097/MD.0000000000046517 (PMC12708221; doi:10.1097/MD.0000000000046517)
Supplement: Supplementary file 1 [file medi-104-e46517-s001.docx]

**Supplementary Table 1. Overview and Summary of the Literature Search**

| **Articles** | 364 |
| --- | --- |
| **Journals** | 140 |
| **Authors** | 1782 |
| **Institutions** | 796 |
| **Countries/Regions** | 58 |
